# Evaluation of the reach and impact of the *100% Jeune *youth social marketing program in Cameroon: findings from three cross-sectional surveys

**DOI:** 10.1186/1742-4755-4-1

**Published:** 2007-02-26

**Authors:** Andrea Plautz, Dominique Meekers

**Affiliations:** 1 Department of International Health and Development, Tulane University School of Public Health and Tropical Medicine, 1440 Canal Street, Suite 2200, New Orleans, LA 70131, USA

## Abstract

**Background:**

The *100% Jeune *youth social marketing program in Cameroon aims to address the high STI/HIV prevalence rates and the high levels of unwanted pregnancy. This study evaluates the *100% Jeune *program, analyzing its reach and impact on condom use, level of sexual activity, and predictors of condom use.

**Methods:**

This analysis uses data from three waves of the Cameroon Adolescent Reproductive Health Survey, implemented at 18-month intervals between 2000 and 2003. The sample is restricted to unmarried youth aged 15–24; sample sizes are 1,956 youth in 2000, 3,237 in 2002, and 3,370 in 2003. Logistic regression analyses determine trends in reproductive health behavior and their predictors, as well as estimate the effect of program exposure on these variables. All regression analyses control for differences in sample characteristics.

**Results:**

A comparison of trends over the 36-month study period shows that substantial positive changes occurred among youth. Results of dose response analyses indicate that some of these positive changes in condom use and predictors of use can be attributed to the *100% Jeune *youth social marketing program. The program contributed to substantial increases in condom use, including consistent use with regular partners among youth of both sexes. Among males, it also contributed to consistent use with casual partners. While condom use increased with both regular and casual partners, levels of use are higher with the latter. Observed secular trends indicate that factors besides the *100% Jeune *program also contributed to the observed improvements. Despite efforts to promote abstinence, the *100% Jeune *program had no effect on levels of sexual activity or number of sexual partners. Likewise, there is no evidence that reproductive health programs for youth lead to increased sexual activity.

**Conclusion:**

Results show that *100% Jeune *successfully used a variety of mass media and interpersonal communication channels to reach a high proportion of youth throughout the intervention period. In a context in which a variety of governmental and nongovernmental partners are increasing youth-focused reproductive health programming, the *100% Jeune *program reached a higher proportion of youth than did other programs. Collective efforts of multiple organizations over time can lead to improvements in adolescent reproductive health. Resources should be allocated to identify and understand predictors of abstinence and partner reduction to inform future programming decisions.

## Background

Human immunodeficiency virus (HIV) rates in Cameroon have increased steadily in the past decade. Although the national HIV prevalence was only 2.9% in 1994, it is now estimated at close to 12% [1-4]. There is evidence that youth in Cameroon are among the populations most at risk for contracting HIV. In 2002, UNAIDS reported an HIV prevalence of 11.5% among pregnant 15–19-year-olds and a 12.2% rate among pregnant 20–24-year-olds. The prevalence of other sexually transmitted infections (STIs), combined with early sexual debut and low rates of condom use, facilitates the spread of HIV among Cameroonian youth [3]. Survey data for the period from 1998 through 2001 estimate the percentage of youth who used a condom at last high-risk sex in the past 12 months at 16% for females aged 15–24 and 31% for males in the same age group [5]. Cameroonian youth also experience high rates of unwanted pregnancies, abortion, and pregnancy-related school dropouts [6-8].

In response to these growing threats to youth in Cameroon, a variety of reproductive health programs are being implemented throughout the country. One such program is *100% Jeune*, a social marketing reproductive health program targeting 15–24-year-olds living in Yaoundé and Douala, the two largest cities in Cameroon. The program uses a combination of mass media and interpersonal communication methods to motivate at-risk urban youth to use condoms consistently or not have sex. This study evaluates the *100% Jeune *program, analyzing its reach and impact on reproductive health outcomes, condom use, level of sexual activity, and predictors of condom use.

### The *100% Jeune *adolescent reproductive health program

With support from the Bill and Melinda Gates Foundation, the *100% Jeune *program was launched in Yaoundé and Douala in 2000 by Programme de Marketing Social au Cameroun (PMSC), an affiliate of Population Services International (PSI). *100% Jeune *aims to motivate at-risk urban youth to engage in healthier sexual behavior and focuses on reducing barriers to condom use. Based on results from formative research (focus group discussions) with youth in Yaoundé and Douala, program messages promote discussion of adolescent reproductive health issues within couples and between youth and their parents, highlight previous sexual history as a risk factor for contracting STIs and HIV, and encourage young females to take responsibility for their reproductive health. The program promotes abstinence and consistent condom use as effective strategies to prevent unwanted pregnancy, STIs, and HIV. Mass media messages focus on increasing condom use with regular partners in particular. All key messages and communications materials were pre-tested before production.

The program uses a variety of mass media and interpersonal communication methods to diffuse its messages and encourage youth to practice and adopt new, healthy behaviors. Peer education sessions and a weekly radio call-in show are key interactive components of the program. Other important elements include a monthly magazine, *100% Jeune, Le Journal*, and a serial radio drama titled *Solange, Let's Talk about Sex*. Integrated television, radio, and billboard campaigns as well as a network of branded youth-friendly *Vendeurs des Amis des Jeunes *condom outlets support these activities. The *100% Jeune *program activities are integrated into a pre-existing national contraceptive social marketing program.

Messages and activities were refined in 2002 on the basis of mid-project research results and an external evaluation of the peer education program [9,10]. New program strategies implemented between the 2002 and 2003 survey waves include encouraging parents to discuss reproductive health issues with their children, making program materials and messages more appealing to females, and integrating participatory approaches into peer education sessions. Less emphasis was placed on promoting youth-friendly condom outlets during the final 18 months of the study period [11].

### Theoretical framework

*100% Jeune *is a theory-based program aiming to reduce barriers to condom use and increase safe behavior–namely abstinence or consistent condom use–among youth. Reproductive health programs often use popular theories such as Social Learning Theory, the Theory of Reasoned Action, and the Health Belief Model to identify behavior change program objectives and activities [12-16]. The *100% Jeune *program is based on a comprehensive theoretical framework that borrows various elements from these behavior change theories [17]. PSI's behavior change framework includes those elements of the popular behavior change theories that were perceived to be amenable to change through social marketing and behavior change programs. The framework assumes that behavior change is a function of a combination of individual, environmental, and social factors, including (1) perceived severity of sexual risks, (2) perceived personal risk (susceptibility), (3) perceived condom attributes and access, (4) perceived social support, and (5) self-efficacy. Recent studies of African youth confirm that these factors influence levels of condom use [18-23], although their relative importance varies across contexts. A recent study of Cameroonian youth also indicates that the determinants of condom use remain constant over time [24]. This analysis measures progress and program impact in each of these five areas that predict condom use.

## Methods

### Data

This analysis uses data from three waves of the Cameroon Adolescent Reproductive Health Survey, implemented at 18-month intervals between 2000 and 2003. The surveys were commissioned by PMSC and implemented by IRESCO (Institut de Recherche et des Études de Comportements) in 2000 and 2002 and by FOCAP (Forum Camerounais de Psychologie) in 2003. The first wave was implemented from July 26 to August 10, 2000, the second from January 4 to January 19, 2002 [25,26], and the third from June 19 to July 16, 2003 [27]. The surveys contain information on 15–24-year-old youth living in the cities of Yaoundé and Douala. All surveys used the same base questionnaire that covered a range of reproductive health topics, including questions regarding HIV risk behavior, level of sexual activity, and condom use. The 2002 and 2003 surveys included additional questions to assess exposure to the *100% Jeune *program and other youth programs. The questionnaires for each of the three survey waves were pretested among a group of 30 male and female youth.

A four-stage stratified sampling design was used for all three surveys. In 2000, the target sample size was 2,000; this figure was increased to 3,500 in 2002 and 2003. The sample was equally divided between Yaoundé and Douala. Using probability of selection proportional to population size (PPS), neighborhoods (*quartiers*) were selected in each city. Twelve neighborhoods were selected for the 2000 survey and 20 were chosen for the 2002 and 2003 surveys. Again using PPS, a total of 30 enumeration areas were chosen in the selected neighborhoods. Households were selected randomly from a list of households in which at least one eligible person resided, using a Kish grid. One eligible person per household was randomly selected and up to three interview attempts were made. No replacements were made for respondents who could not be reached.

Same-sex interviewers, aged 25 or younger, participated in a three-day training workshop prior to conducting the interviews in 2000 and 2002, and interviewers in 2003 received four days of training because of the increased length of the questionnaire. The fieldwork team for the 2000 survey consisted of 24 interviewers and 4 supervisors. The increased survey sample size in 2002 and 2003 required the size of the fieldwork team to be increased to 64 interviewers and 8 supervisors. A substantial number of data collectors worked on more than one of the three surveys. All interviews were conducted in French, the national language, and lasted approximately 45 minutes. Verbal informed consent was obtained from both the head of the household and the respondent. The final sample sizes for the three surveys were 2,096, 3,536 and 3,627, respectively. Our analysis is restricted to the sub-sample of unmarried youth, defined as those who are not currently married or living in union, as this is the main target population for the program. This reduces the effective sample size to 1,956 youth in 2000, 3,237 in 2002, and 3,370 in 2003.

### Measures

#### Program exposure

To measure unprompted exposure to the *100% Jeune *program and other youth reproductive health programs, the following approach was used. Respondents were reminded that reproductive health programs provide family planning, pregnancy, HIV/AIDS prevention, and other services, and that they are implemented by either nongovernmental organizations or government agencies. Youth were then asked to cite all the youth reproductive health programs they have heard of.

In addition, youth were asked prompted questions about their frequency of exposure to specific elements of the *100% Jeune *program. To measure the effect of exposure to the *100% Jeune *program in 2002 and 2003, we computed an indicator based on prompted recall of exposure to various campaign elements. Recent social marketing research has demonstrated that repeated program exposure is needed to achieve substantial changes in health behaviors such as condom use [28]. Therefore, the impact of the program, if any, is expected to be most noticeable among those respondents who had high exposure to each campaign element [29,30]. We defined high exposure the four key elements of the "*100% Jeune*" campaign as follows: 1) often or always listening to the "*100% Jeune*" radio call-in show, 2) having heard at least 10 episodes of the "*Solange*" radio drama, 3) often or always reading "*100% Jeune, le Journal*", and 4) having spoken to a "*100% Jeune*" peer educator. Unfortunately, assessing the net impact of high exposure to each of these campaign elements proved impossible because the exposure variables are correlated. Hence, we created a composite indicator of program exposure. Respondents who reported having high exposure to two or more of the key campaign elements were classified as having "high" exposure, those who reported high exposure to only one campaign element as having "medium-high" exposure, and the remainder as having "low" exposure. We acknowledge that this latter group may include some individuals who had low exposure to several campaign elements, which may result in an underestimation of the level of program impact.

#### Predictors of condom use

As previously described by Meekers et al. [9], we analyzed changes in variables shown to be associated with condom use. The predictor variables cover the following factors in the theoretical framework, described earlier, that was used to design the program. Although the program focuses on these factors to varying degrees, it is necessary to assess all of them to have a full understanding of changes in condom use and reproductive health.

##### - Perceived severity of the health threat

Respondents were asked, "Do you believe that a person who has HIV/AIDS can survive?" and "Do you believe that AIDS can be cured?" The indicators of perceptions of the health threat are two dichotomous variables indicating whether the respondent believes that someone infected with HIV/AIDS can survive and whether she or he believes that AIDS can be cured ("yes" vs. "no/don't know").

##### - Perceived risk

To measure perceived risk of HIV infection, respondents were asked, "If you would not use condoms, would you say your risk of contracting HIV/AIDS would be high, moderate, low, or there would be no risk?" The clause "if you would not use condoms" avoids the problem that some respondents may factor in condom use in their risk assessment while others may not. The variable measures whether respondents believe their risk to be moderate or high versus other. Youth were also asked if they know someone who has HIV/AIDS or who has died of HIV/AIDS ("yes" vs. "no/don't know").

##### - Perceived condom attributes and access

Respondents were asked whether condoms are effective for pregnancy prevention, for STI prevention, and for HIV/AIDS prevention. These two variables are coded as "yes" versus "no/don't know." We also asked youth, "Do you believe that condom use reduces sexual pleasure for men?" Our indicator is a dichotomous variable that equals one if the respondent perceives that condom use reduces sexual pleasure, zero otherwise. Finally, respondents were asked how long it would take to walk from their house to the nearest condom outlet. This information is coded as "knows condom source within 10 minutes" versus "does not know source within 10 minutes or does not know a source at all."

##### - Self-efficacy

Because self-efficacy is known to be a multifaceted concept [31,32], several components of condom use self-efficacy were measured. For this study, respondents' perceived ability to negotiate condom use, to obtain condoms, and to use them correctly are focused on. To measure confidence in their ability to negotiate condom use, interviewers asked respondents who had a regular partner in the past year, "Are you sure that you can convince him/her to use condoms with you (yes vs. no/not sure)?" Respondents were also asked, "Would you be shy buying condoms in a shop near your home?" Our indicator is a dichotomous variable that equals one for respondents who indicated they would not be shy, zero for all others. Finally, to measure whether respondents believe that they have the skills to actually use a condom, respondents were asked, "Are you sure that you know how to correctly use a condom ("yes" vs. "no/not sure")?"

##### - Perceived social support

Youth were asked several questions pertaining to perceived social support. They were asked if their parents support condom use by youth and if their friends are supportive of using condoms. Both questions are coded as "yes" versus "no/don't know." Youth were also asked if they discussed the use of family planning in the past year and whether they discussed STI/AIDS prevention in the past year ("yes" vs. "no/don't know").

#### Sexual activity indicators

Indicators of level of sexual activity include "had two or more sexual partners in the past year" and "had sex in the past year" (defined as having one or more sexual partners in the past year and measured among all respondents).

#### Condom use indicators

The question, "Have you ever used a condom?" was asked to measure the extent to which youth tried condoms. As youth who try condoms do not necessarily continue to use them, we also measured condom use in the last sexual act with a regular partner and use in the last sexual act with a casual partner. These indicators are coded as yes/no. Respondents were also asked about frequency of condom use with their regular and casual partners (never, sometimes, often, or always). These two variables measure consistent condom use and are recoded as "always used condoms" versus "often/sometimes/never used condoms."

#### Reproductive health indicators

Sexually experienced males were asked if they had any STI symptoms in the past year. This variable is coded as "yes" versus "no/don't know." This information was not collected from females, as STIs in females are often asymptomatic.

#### Control variables

To account for confounding factors, control variables include age, city of residence, level of education, school enrollment status, socioeconomic status, number of sexual partners, and spontaneous recall of other adolescent reproductive health programs.

### Methods for data analysis

Logistic regression analyses examine trends in predictors of condom use as well as indicators of condom use and level of sexual activity. To facilitate interpretation, the results of the logistic regression models are presented as adjusted proportions. The adjusted proportions are the expected proportions of the outcome variable after controlling for differences in the background variables. Thus, the adjusted proportions show the levels of the outcome variable for each category of our exposure variable, after controls  [33,34][35]. The trend analyses of indicators of sexual activity control for age, city of residence, level of education, school enrollment status and socioeconomic status. All other trends analyses also control for the number of sexual partners the respondent had in the previous year. As previous studies have shown that the impact of adolescent reproductive health programs in Cameroon varies by gender [36,37]. all analyses are conducted separately for male and female respondents.

In a subsequent section, logistic regression analyses also study how predictors of condom use, level of sexual activity, condom use, and reproductive health outcomes vary with level of exposure to the *100% Jeune *program. These dose response analyses measure to what extent youth with medium and high program exposure in 2002 and 2003 have better results than those with low program exposure in 2002 (the reference group) after controlling for age, city of residence, level of education, school enrollment status, socioeconomic status, and spontaneous recall of other programs. As with the trend analyses, all analyses (except those where the outcome pertains to having multiple partners) also control for number of sexual partners. Comparison of youth with low program exposure in 2002 and 2003 provides an estimate of secular trends not attributable to the program. These analyses are also estimated separately for male and female respondents.

## Sample description

Table 1 shows the characteristics of the study samples. For all three surveys, half of the youth reside in the city of Douala and half in Yaoundé, as specified by the study design. Results show that both the 2002 and 2003 survey populations are younger than respondents in 2000. However, the 2002 and 2003 survey populations do not differ with respect to age. While there are more males than females in each survey round, the gender distribution remains the same across surveys (approximately 55% male and 45% female). Results from the 2001 Demographic and Health Survey also show a higher percentage of males in the youth population. The 2000 and 2003 survey populations are similar in terms of the level of education attained–about 90% of youth reached at least a secondary school education–whereas the 2002 survey population is less well educated, with only 85% having a secondary school education. The three samples also differ in terms of the percentage of youth in school and socioeconomic status. These differences between the study populations are controlled for in the analysis.

**Table 1:** Sample Characteristics

	**Survey Wave**			**Significance**		
	**2000**	**2002**	**2003**	**2000 vs. 2002**	**2002 vs. 2003**	**2000 vs. 2003**
	
**Age**				***		**
15–19	57.3	61.1	60.3			
20–24	42.7	38.9	39.7			
**Gender**						
Male	54.0	54.2	55.0			
Female	46.0	45.8	45.0			
**Education**				***	***	
Secondary +	90.1	84.7	91.4			
< Secondary	9.9	15.3	8.6			
**Student**				***	***	***
No	33.6	42.7	27.8			
Yes	66.4	57.3	72.2			
**Socioeconomic Status**				***	***	***
Low	27.6	31.6	28.7			
Medium	38.1	42.5	45.3			
High	34.3	25.9	26.1			
**City**						
Yaoundé	50.9	48.9	49.3			
Douala	49.1	51.1	50.7			
**Number of Sexual Partners**				***	***	
None	42.2	37.8	41.6			
One	31.6	31.7	33.4			
Two or More	26.3	30.5	25.0			
**Number of Regular Partners**				***	***	
None	47.8	41.9	45.9			
One	36.4	36.1	37.8			
Two or More	15.8	22.0	16.2			
**Number of Casual Partners**						
None	81.6	82.4	83.1			
One	8.1	7.4	7.7			
Two or More	10.3	10.4	9.3			

**Number of Cases**	1,956 (100%)	3,237 (100%)	3,370 (100%)			

*** p < .01; ** p < .05.

Data on the self-reported number of sexual partners show that the percentage of youth who reported having two or more partners increased from 26% in 2000 to 31% in 2002, but then decreased again to 25% in 2003. Breakdown by partner type further shows that these changes are caused by changes in the number of regular partners, rather than casual partners.

## Results

### Reach of *100% Jeune *and other youth reproductive health programs

Examination of unprompted questions about program exposure shows that the percentage of youth reporting having heard of at least one program increased significantly throughout the study period, from 35% in 2000 to 42% in 2002 and to 50% in 2003 (not shown). Unprompted recall of *100% Jeune *increased dramatically from 1% in 2000 to 26% in 2002. Between 2002 and 2003, the level of unprompted recall remained constant. Only the *Cercle des Amis du Cameroun *(CERAC) program (19%), *Entre Nous Jeunes *(9%), and the program conducted by the Ministère de la Santé Publique (9%) had unprompted recall higher than 5% in 2003. It is noted, however, that exposure to any program increased little between 2000 and 2002 despite the high reach of *100% Jeune*. This suggests that the program predominantly reached youth who had already been reached by other programs.

Prompted recall of specific *100% Jeune *program elements is shown in Table 2. Results are limited to the 2002 and 2003 survey rounds, as the 2000 survey occurred before the *100% Jeune *program was fully implemented. Table 2 shows that recall of several program elements was higher in 2003 than in 2002. Similarly, the percentage of youth reporting high levels of exposure to these program elements was higher at the time of the 2003 survey than in the 2002 survey. Approximately 12% of youth in 2002 and 2003 reported ever attending a peer education session, while the percentage who ever spoke to a peer educator increased slightly but significantly, from 8% to 11% between 2002 and 2003. Reach of the *100% Jeune, Le Journal *magazine, the *100% Jeune *radio call-in show, and the *Solange, Let's Talk about Sex *radio drama also increased. About three-quarters of all youth (74%) in 2002 reported having read the magazine at least once, compared with 90% in 2003. The level of exposure to the magazine also increased significantly between 2002 and 2003. The percentage of youth who report reading it often or always increased from 34% to 48%. The reach of the radio call-in show increased from 47% to 52% of youth between the last two surveys, and at the time of the 2003 survey, 32% of youth had listened to the *Solange, Let's Talk about Sex *radio drama at least once in the previous three months, compared with 26% in 2002.

**Table 2:** Percentage of Youth Who Recall Exposure to Various *100% Jeune *Campaign Elements (prompted recall, 2002 and 2003 survey waves only)

	**Survey Wave**		
		
	**2002**	**2003**	
**Campaign Element**	**(%)**	**(%)**	**Significance**

**Peer Education and Promotion**			
Ever attended peer education session	11.9	12.3	
Ever talked to peer educators	7.6	10.6	***
			
***100% Jeune, Le Journal *Magazine**			
Ever read *100% Jeune *magazine	73.9	89.8	***
How often do you read the magazine?			***
- Never	26.2	10.2	
- Sometimes	39.7	42.3	
- Often	18.3	28.4	
- Always	15.9	19.1	
			
***100% Jeune *Radio Call-In Show**			
Heard *100% Jeune *radio program in past 3 months	47.3	51.5	***
How often do you usually listen?			***
- Never	52.7	48.5	
- Sometimes	31.1	34.9	
- Often	11.2	13.7	
- Always	5.1	2.8	
			
***Solange, Let's Talk about Sex *Radio Drama**			
Heard *Solange *radio series in past 3 months	26.0	31.5	***
How many different episodes have you heard?			***
- None	74.0	68.5	
- One to four	13.9	18.9	
- Five to nine	6.3	8.2	
- Ten or more	5.8	4.4	
			
**Mass Media Campaign**			
Saw TV spot in past 3 months	70.7	69.9	
Heard radio spot in past 3 months	64.5	58.9	***
			
**Youth-Friendly Vendors**			
Heard about Vendeurs Amis des Jeunes outlets in past 3 months	31.9	19.7	***
Visited a Vendeurs Amis des Jeunes outlet in past 3 months	8.8	4.5	***

**Number of Cases**	3,237	3,370	

*** p < .01.

In terms of exposure to mass media spots, about 70% of youth in both 2002 and 2003 reported seeing a television spot in the past three months, but exposure to radio spots decreased from 65% in 2002 to 59% in 2003. Exposure to youth-friendly condom vendors also decreased between 2002 and 2003. Only 20% of youth in 2003 reported hearing of them in the past three months, compared with 32% in 2002. The percentage of youth visiting a youth-friendly vendor also decreased from 9% to 5% between the two survey rounds.

### Trends in condom use, level of sexual activity, and predictors of condom use

Table 3 and Table 4 illustrate trends in condom use, level of sexual activity, and predictors of condom use, after controlling for other factors. We tested the significance of trends observed between each survey wave (2000 vs. 2002, 2002 vs. 2003) as well as across the entire study period (2000 vs. 2003). Table 3 shows results for females and Table 4 shows results for males.

**Table 3:** Logistic Regression Results of Trends in Predictors of Condom Use and Trends in Indicators of Condom Use, Adjusted Percentages among Females

	**Females**			**Significance**		
	
	**2000**	**2002**	**2003**	**2000 vs. 2002**	**2002 vs. 2003**	**2000 vs. 2003**
**Perceived Severity**						
-HIV-positive person can survive	14.3	35.5	50.9	***	***	***
-AIDS can be cured	10.9	17.2	17.2	***		***

**Perceived Risk**						
-Moderate/high personal risk	51.7	65.7	57.5	***	***	***
-Knows someone who has/died of HIV/AIDS	45.0	45.8	47.6			

**Perceived Condom Attributes and Access**						
-Condoms effective for FP	75.5	72.5	85.2		***	***
-Condoms effective for HIV/AIDS prevention	89.8	88.7	93.6		***	***
-Condom source within 10 minutes	62.1	68.1	74.0	***	***	***
-Condoms reduce sexual pleasure	36.0	39.1	30.1		***	***

**Self-Efficacy**						
- Can convince regular partner to use condoms ^a^	80.5	83.0	89.9		***	***
- Can convince casual partner to use condoms ^b^	86.1	83.3	92.4		**	
- Not shy to obtain condoms in nearby shop	42.7	55.3	57.4	***		***
- Confident knows correct condom use	38.3	46.4	53.1	***	***	***

**Perceived Social Support**						
-Friends support youth condom use	74.0	81.1	86.4	***	***	***
-Parents support youth condom use	58.4	68.0	71.3	***		***
-Discussed family planning with friends in past year	17.8	21.8	29.4		***	***
-Discussed family planning with others in past year	28.0	25.4	33.8		***	**
-Discussed STI/AIDS with friends in past year	32.5	39.2	44.3	**	**	***
-Discussed STI/AIDS with others in past year	38.6	31.7	45.2	***	***	**

**Sexual Activity**						
-Had sex in the past year	71.5	74.1	74.4			
-Had 2+ sexual partners in the past year	15.1	19.3	13.2	***	***	

**Condom Use**						
-Ever used condoms	50.0	61.6	64.9	***		***
-Used condom in last sex with regular partner ^a^	32.9	46.0	62.1	***	***	***
-Always uses condoms with regular partner ^a^	13.7	21.0	31.4	***	***	***
-Used condom in last sex with casual partner ^b^	46.1	65.5	84.1	**	***	***
-Always uses condoms with casual partner ^b^	29.1	49.6	69.9	***	***	***

**Reproductive Health**						
**-**Had an STI symptom in the past year	n.a.	n.a.	n.a.			

**Number of Cases (100%)**	899	1,483	1,518			

*** p < .01; ** p < .05. FP = family planning; HIV = human immunodeficiency virus; STI/AIDS = sexually transmitted infections/acquired immunodeficiency syndrome.

Analysis of sexual activity control for age, city of residence, level of education, school enrollment, and socioeconomic status. All other analyses also control for number of sexual partners.

^a ^N = 474, 902, and 848, respectively.

^b ^N = 76, 105, and 115, respectively.

**Table 4:** Logistic Regression Results of Trends in Predictors of Condom Use and Trends in Indicators of Condom Use, Adjusted Percentages among Males

	**Males**			**Significance**		
	
	**2000**	**2002**	**2003**	**2000 vs. 2002**	**2002 vs. 2003**	**2000 vs. 2003**
**Perceived Severity**						
-HIV-positive person can survive	25.2	38.9	42.2	***	**	***
-AIDS can be cured	21.1	22.6	21.6			

**Perceived Risk**						
-Moderate/high personal risk	73.4	73.8	59.3		**	***
-Knows someone who has/died of HIV/AIDS	36.5	32.6	39.7	**	***	

**Perceived Condom Attributes and Access**						
-Condoms effective for FP	79.4	82.9	87.5	**	***	***
-Condoms effective for HIV/AIDS prevention	88.0	87.8	90.8		***	**
-Condom source within 10 minutes	80.0	84.1	90.4	***	***	***
-Condoms reduce sexual pleasure	55.5	54.1	45.0		***	***

**Self-Efficacy**						
- Can convince regular partner to use condoms ^a^	86.8	89.9	92.9		**	***
- Can convince casual partner to use condoms ^b^	89.0	90.1	93.8		**	**
- Not shy to obtain condoms in nearby shop	61.1	68.1	78.1	***	***	***
- Confident knows correct condom use	65.9	71.9	71.9	***		***

**Perceived Social Support**						
-Friends support youth condom use	83.0	85.0	88.7		***	***
-Parents support youth condom use	64.3	74.8	75.6	***		***
-Discussed family planning with friends in past year	27.7	28.5	36.9		***	***
-Discussed family planning with others in past year	37.1	26.2	35.9	***	***	
-Discussed STI/AIDS with friends in past year	49.4	59.0	62.8	***		***
-Discussed STI/AIDS with others in past year	46.7	36.8	49.3	***	***	

**Sexual Activity**						
-Had sex in the past year	80.4	80.8	82.4			
-Had 2+ sexual partners in the past year	31.9	35.3	32.7			

**Condom Use**						
-Ever used condoms	58.7	64.9	70.2	**	***	***
-Used condom in last sex with regular partner ^a^	44.3	61.5	75.7	***	***	***
-Always uses condoms with regular partner ^a^	19.5	33.2	44.8	***	***	***
-Used condom in last sex with casual partner ^b^	61.0	68.7	81.3	**	***	***
-Always uses condoms with casual partner ^b^	45.3	52.5	70.1		***	***

**Reproductive Health**						
**-**Had an STI symptom in the past year ^c^	19.6	14.7	10.7	***	***	***

**Number of Cases (100%)**	1,057	1,754	1,852			

*** p < .01; ** p < .05. FP = family planning; HIV = human immunodeficiency virus; STI/AIDS = sexually transmitted infections/acquired immunodeficiency syndrome.

Analysis of sexual activity control for age, city of residence, level of education, school enrollment, and socioeconomic status. All other analyses also control for number of sexual partners.

^a ^N = 541, 978, and 974, respectively.

^b ^N = 282, 464, and 456, respectively.

^c ^Among sexually experienced males only. N = 721, 1,247, and 1,220, respectively.

Among both males and females, almost all predictors of condom use improved during the study period, as did the indicators of condom use. However, levels of risky sexual activity did not decline. Among males, self-reported STI symptoms decreased. Below, these findings are described in detail.

#### Perceived severity

Youth's perception of the severity of HIV and AIDS decreased over the three-year period. Among both sexes, youth in 2003 were more likely than they were in 2000 to feel that an HIV-positive person can survive. Among females, the percentage believing that an HIV-positive person could survive increased from 14% in 2000 to 51% in 2003; among males, the percentage increased from 25% to 42%. Female youth in 2002 and 2003 were also more likely than female youth in 2000 to report that AIDS can be cured. These perceptions may reflect an increased awareness of antiretroviral therapies that are prolonging the lives of those infected with HIV.

#### Perceived risk

Trends in youth's perception of their perceived personal risk for HIV are mixed. Among females, the percentage reporting a moderate to high perceived risk for HIV infection increased from 52% in 2000 to 66% in 2002, but then declined to 58% in 2003. Among males, the corresponding percentage was constant at roughly 73% in 2000 and 2002, but then declined significantly to 59% in 2003.

#### Perceived condom attributes and access

Youth's knowledge of where to obtain condoms and their opinions about them improved over the 36-month study period. Between 2002 and 2003, the percentage reporting knowledge of a condom source within a 10-minute walk increased from 62% to 74% among females, and from 80% to 90% among males. Although the vast majority of both female and male youth in 2000 reported that condoms are effective for family planning and effectively prevent HIV, positive trends are observed for both indicators between 2000 and 2003. The percentage of females stating that condoms are effective for family planning increased from 76% to over 85%; for males, it increased from 76% to 88%. Similar results are observed regarding the effectiveness of condoms for HIV prevention. Most of these improvements occurred between the 2002 and 2003 surveys. Youth of both sexes are also less likely to report that condoms reduce sexual pleasure in 2003 than in 2000.

#### Self-efficacy

Results from the three surveys indicate that over time, Cameroonian youth generally became more comfortable buying condoms and negotiating condom use, and also gained confidence about correct condom use. The percentage of females who are not shy about buying condoms in a nearby store increased from 43% in 2000 to 57% in 2003; among males it increased from 61% to 78%. Although levels were high at baseline–over 80%–both females and males reported more frequently in 2003 than in previous years that they were able to convince their regular partners to use condoms. Confidence about knowing how to use condoms correctly also increased, from 38% to 53% among females and from 66% to 72% among males across the three survey waves.

#### Social support

Social support for youth condom use increased. Youth are more likely to report that their friends support youth condom use (86% in 2003 vs. 74% in 2000 among females, and 89% vs. 83% among males). The percentage of youth stating that their parents support condom use also rose between 2000 and 2003. In addition, respondents more frequently discuss family planning as well as STI/AIDS in the past year.

#### Sexual activity

No changes are noted across the three surveys in the percentage of youth who report having had sex in the past year–about 75% of female youth and 80% of males reported being sexually active in the past year. The percentage of female youth who reported having two or more sexual partners in the year before the survey increased from 15% in 2000 to 19% in 2002. However, between 2002 and 2003 the percentage decreased significantly from 19% to 13%. This latter percentage is not significantly different from the percentage observed at the 2000 baseline survey. Among males, no significant changes in the number of sexual partners were observed.

#### Condom use

Table 3 and Table 4 show that among both male and female youth, all measures of condom use improved steadily over time. Youth became more likely to report using a condom during their last sexual intercourse with a regular partner as well as with a casual partner. Specifically, the percentage of females who reported using a condom during their last intercourse with their regular partner increased from 33% in 2000 to 46% in 2002 and to 62% in 2003. For males, the corresponding percentages are 44%, 62%, and 76%, respectively. Similar increases were observed in consistent condom use, with both regular and casual partners. Consistent use with regular partners increased from 14% in 2000 to 21% in 2002 and to 31% in 2003 among females, and from 20% in 2000 to 33% in 2002 and to 45% in 2003 among males. Among females, consistent condom use with casual partners increased from 29% to 50% during the first phase of the project (between the 2000 and 2002 surveys) and from 50% to 70% during the second phase (between the 2002 and 2003 surveys). Among males, consistent use with casual partners increased from 45% to 53% during the first phase, and further increased to 70% by the time of the 2003 survey.

#### Reproductive health

In terms of reproductive health outcomes, reported STIs declined over time. The percentage of sexually experienced males reporting an STI symptom in the past year decreased steadily, from 20% in 2000 to 11% in 2003.

### The effect of exposure to the *100% Jeune *program

Table 5 and Table 6 examine to what extent reproductive health outcomes, level of sexual activity, condom use, and predictors of condom use vary with level of exposure to the *100% Jeune *program in 2002 and 2003. Because program activities were not fully implemented at the time of the 2000 survey, the analysis is limited to the latter two survey waves. To measure the impact of program exposure, we conduct logistic regression analysis with a six-category exposure variable (representing low, medium, high program exposure in each of the two survey years) as a predictor. We then compare the outcome measures each exposure category with those for youth who reported having low exposure in the 2002 survey, who serve as the reference group.

**Table 5:** Logistic Regression Results of Level of Exposure to the *100% Jeune *program on Outcome Measures, Adjusted Percentages among Females

	**2002**			**2003**		
	
	**Low exposure (ref.)**	**Medium-high exposure**	**High exposure**	**Low exposure**	**Medium-high exposure**	**High exposure**
**Perceived Severity**						
-HIV-positive person can survive	32.7	38.1	37.7	48.2***	53.6***	51.5***
-AIDS can be cured	17.5	17.6	14.0	18.8	16.0	15.8

**Perceived Risk**						
-Moderate/high personal risk	64.0	65.7	70.9	55.6***	59.6	56.7**
-Knows someone who has/died of HIV/AIDS	41.0	50.0***	52.0***	45.9	50.4***	44.0

**Perceived Condom Attributes and Access**						
-Condoms effective for FP	72.4	72.4	73.3	84.1***	84.6***	89.0***
-Condoms effective for HIV/AIDS prevention	88.4	88.4	60.6	93.6***	94.2***	93.7**
-Condom source within 10 minutes	65.0	69.2	74.4**	72.2***	77.2***	71.9**
-Condoms reduce sexual pleasure	40.5	39.0	31.9**	33.6***	30.1***	23.3***

**Self-Efficacy**						
- Can convince regular partner to use condoms ^a^	82.4	82.7	86.9	89.0***	90.8***	90.6**
- Can convince casual partner to use condoms ^b^	–	–	–	–	–	–
- Not shy to obtain condoms in nearby shop	51.9	55.6	65.8***	51.9	60.0***	65.0***
- Confident knows correct condom use	38.1	50.1***	64.5***	47.6***	55.5***	59.4***

**Perceived Social Support**						
-Friends support youth condom use	78.3	82.3	85.8**	84.5***	87.2***	88.1***
-Parents support youth condom use	66.6	68.7	69.0	67.6	74.8***	71.1
-Discussed family planning with friends in past year	16.8	27.5***	25.3**	27.6***	24.6***	38.7***
-Discussed family planning with others in past year	23.4	23.9	33.0**	28.6	31.2**	47.8***
-Discussed STI/AIDS with friends in past year	35.2	43.3**	46.9**	42.0**	43.0**	49.1***
-Discussed STI/AIDS with others in past year	28.8	31.6	39.8**	41.7***	41.6***	58.9***

**Sexual Activity**						
-Had sex in the past year	77.8	75.7	77.2	75.5	77.5	80.4
-Had 2+ sexual partners in the past year	17.7	22.0	19.3	13.8**	12.8**	12.8

**Condom Use**						
-Ever used condoms	61.4	62.2	67.3	64.2	67.9**	67.5
-Used condom in last sex with regular partner ^a^	47.3	42.2	50.0***	60.8***	60.0***	68.5***
-Always uses condoms with regular partner ^a^	20.2	20.8	24.8	26.7**	32.6***	39.2***
-Used condom in last sex with casual partner ^b^	–	–	–	–	–	–
-Always uses condoms with casual partner ^b^	–	–	–	–	–	–

**Reproductive Health**						
**-**Had an STI symptom in the past year ^c^	–	–	–	–	–	–

**Number of Cases (100%)**	789	494	200	640	589	289

*** p < .01; ** p < .05. FP = family planning; HIV = human immunodeficiency virus; STI/AIDS = sexually transmitted infections/acquired immunodeficiency syndrome.

Analyses of sexual activity control for age, city of residence, level of education, school enrollment status, socioeconomic status, and spontaneous recall of other programs. All other analyses also control for number of sexual partners.

^a ^N = 519, 275, and 108 in 2002 and 371, 314, and 163 in 2003.

^b ^Results are omitted because of the small number of cases.

^c ^Among sexually experienced males only because STIs are often asymptomatic among females. N = 686, 367, and 194 in 2002 and 624, 389, and 207 in 2003.

**Table 6:** Logistic Regression Results of Level of Exposure to the *100% Jeune *program on Outcome Measures, Adjusted Percentages among Males

	**2002**			**2003**		
	
	**Low exposure (ref.)**	**Medium-high exposure**	**High exposure**	**Low exposure**	**Medium-high exposure**	**High exposure**
**Perceived Severity**						
-HIV-positive person can survive	39.6	37.9	37.5	42.1	42.3	42.0
-AIDS can be cured	23.7	21.2	20.9	22.0	21.2	21.5

**Perceived Risk**						
-Moderate/high personal risk	71.4	77.7**	78.7**	55.7***	63.6***	61.5***
-Knows someone who has/died of HIV/AIDS	30.0	35.9**	35.3	35.6**	44.9***	42.0***

**Perceived Condom Attributes and Access**						
-Condoms effective for FP	81.8	83.0	86.1	86.1**	88.7***	89.8***
-Condoms effective for HIV/AIDS prevention	86.6	90.0	88.4	90.4***	92.2***	89.8
-Condom source within 10 minutes	82.4	86.9**	83.8	89.7***	92.1***	88.9***
-Condoms reduce sexual pleasure	53.1	57.5	51.4	49.2	43.7***	35.9***

**Self-Efficacy**						
- Can convince regular partner to use condoms ^a^	87.9	92.5**	93.9**	92.1**	93.8***	93.5
- Can convince casual partner to use condoms ^b^	87.7	91.3	97.0**	93.8**	92.7	95.5
- Not shy to obtain condoms in nearby shop	64.8	73.8***	70.0	75.3***	82.1***	79.3***
- Confident knows correct condom use	67.9	76.8***	75.9**	66.3	77.5***	77.2***

**Perceived Social Support**						
-Friends support youth condom use	83.2	87.4**	89.0**	86.3	91.5***	90.5***
-Parents support youth condom use	73.0	76.5	77.1	71.0	80.9***	78.9**
-Discussed family planning with friends in past year	25.5	29.5	36.1***	39.1***	32.0**	36.5***
-Discussed family planning with others in past year	22.5	28.8**	32.8***	34.7***	32.6***	40.5***
-Discussed STI/AIDS with friends in past year	54.6	64.8***	67.1***	63.5***	60.9**	63.0**
-Discussed STI/AIDS with others in past year	31.8	38.6**	49.7***	44.1***	50.7***	58.6***

**Sexual Activity**						
-Had sex in the past year	79.4	81.5	84.3	81.7	83.1	83.9
-Had 2+ sexual partners in the past year	33.8	37.2	36.2	32.7	30.5	35.7

**Condom Use**						
-Ever used condoms	60.9	71.7***	69.6**	68.2**	73.9***	75.9***
-Used condom in last sex with regular partner ^a^	57.7	62.8	73.0***	70.2***	80.8***	81.6***
-Always uses condoms with regular partner ^a^	29.4	35.1	39.5**	37.7***	52.1***	52.7***
-Used condom in last sex with casual partner ^b^	66.1	76.2**	65.5	77.8***	85.6***	89.3***
-Always uses condoms with casual partner ^b^	48.0	64.7***	45.1	63.4***	79.9***	79.3***

**Reproductive Health**						
**-**Had an STI symptom in the past year ^c^	16.1	12.8	12.0	11.2***	8.4***	11.6

**Number of Cases (100%)**	976	504	274	929	601	322

*** p < .01; ** p < .05. FP = family planning; HIV = human immunodeficiency virus; STI/AIDS = sexually transmitted infections/acquired immunodeficiency syndrome.

Analyses of sexual activity control for age, city of residence, level of education, school enrollment status, socioeconomic status, and spontaneous recall of other programs. All other analyses also control for number of sexual partners.

^a ^N = 532, 282, and 164 in 2002 and 483, 314, and 177 in 2003.

^b ^N = 261, 138, and 65 in 2002 and 249, 139, and 68 in 2003.

^c ^Among sexually experienced males only. N = 686, 367, and 194 in 2002 and 624, 389, and 207 in 2003.

To measure the extent of secular trends occurring independently of exposure to the *100% Jeune *program, we compared youth with low exposure in the 2003 with the reference group (low exposure in 2002). If a secular trend exists that cannot be attributed to the *100% Jeune *program, we expect that even youth with low program exposure will show significant improvements between 2002 and 2003. If the *100% Jeune *program contributed to observed improvement, we expect that youth with medium-high and/or high exposure in 2003 will have significantly better results for a variable than those in the reference group, and also that these results will be substantially better than any observed secular trend.

As before, the results of the logistic regression analyses are shown as adjusted percentages. The analyses of indicators of sexual activity have been controlled for age, city of residence, level of education, school enrollment status, socioeconomic status, and spontaneous recall of other reproductive health programs. The analyses for all other outcome measures have also been controlled for number of sexual partners. Table 5 shows results among females and Table 6 reports results among males.

The results in Table 5 and Table 6 indicate that improvement occurred for a variety of indicators. Among females reporting high exposure to the *100% Jeune *program in the 2003 survey, 39% report consistently using condoms with regular partners, compared with 20% among those in the 2002 low-exposure reference group. In contrast, the secular trend indicates improvement from 20% to only 27%. Among males, 53% of those with high exposure in 2003 report consistent condom use with their regular partners, compared with only 29% of those in the reference group. The secular trend among males shows improvement from 29% to only 38%. Among males, the program also contributed to improvements in condom use in the last sexual act with a casual partner and consistent use with a casual partner; 79% of male youth with high exposure in 2003 reported consistent condom use with a casual partner, compared with 48% among those in the reference group. In comparison, the secular trend indicated an improvement from 48% to 63%. Condom use among females could not be assessed because of the small number of females reporting casual partners.

Between 2002 and 2003, the program also appears to have contributed to improvements in predictors of condom use, including perceived condom attributes, perceived social support, and self-efficacy among youth of both sexes. Among females, the data indicate that the program reduced shyness in purchasing condoms and increased discussion of family planning with people other than their friends. Among males, high program exposure in 2003 is associated with peer support of condom use, and no secular trend is noted. In addition, among both sexes, high program exposure is also associated with the belief that parents support condom use.

The program did not decrease the level of sexual activity or reduce the number of sexual partners, despite efforts to promote abstinence. Furthermore, it did not affect the percentage of males reporting STI symptoms.

For several variables, there were observed secular trends not attributable to the *100% Jeune *program. This indicates that factors besides the *100% Jeune *program also contributed to improvements in outcome variables. For example, among females with low program exposure, the percentage who believe condoms are effective for family planning increased significantly, from 72% in 2002 to 84% in 2003. Among males, the percentage for this indicator increased from 82% to 86% between 2002 and 2003. Secular trends were also observed in the areas of perceived condom effectiveness, condom access, self-efficacy, social support, and condom use.

## Conclusion

This paper examines the *100% Jeune *adolescent reproductive health program implemented in the cities of Douala and Yaoundé, Cameroon. The *100% Jeune *program targets 15–24-year-old youth using a combination of mass media and interpersonal communication channels to motivate them to use condoms consistently or not have sex. This study analyzes the program's reach and its impact on condom use, level of sexual activity, and predictors of condom use. The results are based on three rounds of an adolescent reproductive health survey conducted at 18-month intervals between 2000 and 2003.

Results show that *100% Jeune *successfully used a variety of mass media and interpersonal communication channels to reach a high proportion of youth throughout the intervention period. About one in four youth (26% and 28%, respectively) surveyed in both 2002 and 2003 spontaneously recalled the *100% Jeune *program. In a context in which a variety of governmental and nongovernmental partners are increasing youth-focused reproductive health programming, the *100% Jeune *program reached a higher proportion of youth than did other programs. In addition, the percentage of youth who recalled specific *100% Jeune *program elements was higher in the 2003 survey than in the 2002 survey. In the 2003 survey, youth were also more likely to report repeated exposure to campaign elements than was the case in 2002. Television spots and the monthly magazine reached the highest percentage of youth. Although only 12% of all youth were reached through peer education, the percentage of youth who reported speaking with a program peer educator was higher in 2003 than in the 2002 survey. This result may be related to the program's increased efforts to integrate participatory approaches into peer education sessions.

An examination of trends in behaviors and related predictors over the 36-month study period shows that substantial positive changes occurred among youth. Results of dose response analyses indicate that some of these positive changes can be attributed to the *100% Jeune *youth social marketing program. The program contributed to substantial increases in condom use, including consistent use with regular partners among youth of both sexes. Among males, it also contributed to consistent use with casual partners. Whereas the program had some impact on condom use among males after the first 18 months of activities, the 2003 results reflect increased consistent condom use among both females and males with high exposure to the *100% Jeune *program. Results also indicate that the *100% Jeune *program contributed to important improvements in predictors of condom use. The program did not decrease the level of sexual activity or reduce the number of sexual partners, despite efforts to promote abstinence. Furthermore, it did not affect the percentage of males reporting STI symptoms.

In addition, the observed secular trends indicate that factors besides *100% Jeune *also contributed to the improvement observed in the areas of perceived condom effectiveness, condom access, self-efficacy, social support, and condom use. These results indicate that collective efforts of multiple organizations over time can lead to improvements in adolescent reproductive health.

Although there are signs of increasing use of condoms and reduced barriers among Cameroonian youth, several challenges remain. Youth of both sexes continue to believe they are not at risk of contracting HIV/AIDS, and social barriers continue to limit open discussion of reproductive health issues. Females still appear to be less likely than males to believe they can buy and use condoms correctly. Future programs should aim to address these and other barriers to improved adolescent reproductive health. Programs should devote resources to motivating youth to abstain, or reduce numbers of sexual partners, and continue to encourage sexually active youth to use condoms consistently, that is, with every partner, every time. Resources should be allocated to identify and understand predictors of abstinence and partner reduction to inform future programming decisions.

## Study limitations

This study is subject to several limitations. As with all studies based on cross-sectional data, the direction of causal relationships cannot always be determined. However, the three survey waves provide information about the temporal order of events, which in turn can help establish causality. Also, behavioral outcomes are self-reported and therefore subject to reporting errors and biases. Finally, although information on spontaneous recall was collected for all programs, it was more difficult to obtain information about specific program elements for reproductive health programs other than *100% Jeune*. This difficulty limits the extent to which we can control for exposure to other programs.

## Competing interests

At the time of writing, Miss Plautz was Research Associate with Population Services International (PSI), which is the mother company of the organization that implemented the program that is being assessed in this study. Dr. Meekers is the former Research Director of PSI, and was a paid consultant on this project.

## Authors' contributions

AP conducted the statistical analyses and drafted the manuscript. DM developed the study design, assisted with the analysis, and contributed to the writing of the final manuscript. Both authors read and approved the final manuscript.
